# Monitoring PSA levels as chemical state-variables in metal-oxide memristors

**DOI:** 10.1038/s41598-020-71962-3

**Published:** 2020-09-17

**Authors:** Ioulia Tzouvadaki, Spyros Stathopoulos, Tom Abbey, Loukas Michalas, Themis Prodromakis

**Affiliations:** Centre for Electronics Frontiers, Zepler Institute for Photonics and Nanoelectronics, University of Southampton, Southampton, SO17 1BJ UK

**Keywords:** Cancer, Biomarkers, Health care, Medical research, Engineering, Materials science, Nanoscience and technology

## Abstract

Medical interventions increasingly rely on biosensors that can provide reliable quantitative information. A longstanding bottleneck in realizing this, is various non-idealities that generate offsets and variable responses across sensors. Current mitigation strategies involve the calibration of sensors, performed in software or via auxiliary compensation circuitry thus constraining real-time operation and integration efforts. Here, we show that bio-functionalized metal-oxide memristors can be utilized for directly transducing biomarker concentration levels to discrete memory states. The introduced chemical state-variable is found to be dependent on the devices’ initial resistance, with its response to chemical stimuli being more pronounced for higher resistive states. We leverage this attribute along with memristors’ inherent state programmability for calibrating a biosensing array to render a homogeneous response across all cells. Finally, we demonstrate the application of this technology in detecting Prostate Specific Antigen in clinically relevant levels (ng/ml), paving the way towards applications in large multi-panel assays.

## Introduction

Memristive technologies^1–3^ hold great potential for delivering highly scalable^4^, power-efficient^5,6^ electronic systems, while exhibiting more functionalities such as multiple resistive states (RS)^7^ and reconfigurability^8^. So far, the focus of memristors implementation has mainly been devoted on memory-oriented and neuromorphic applications^9–12^, with some more recent uses of the technology being exploited for linking biological functions with engineering systems^13–15^.

**Figure 1:**
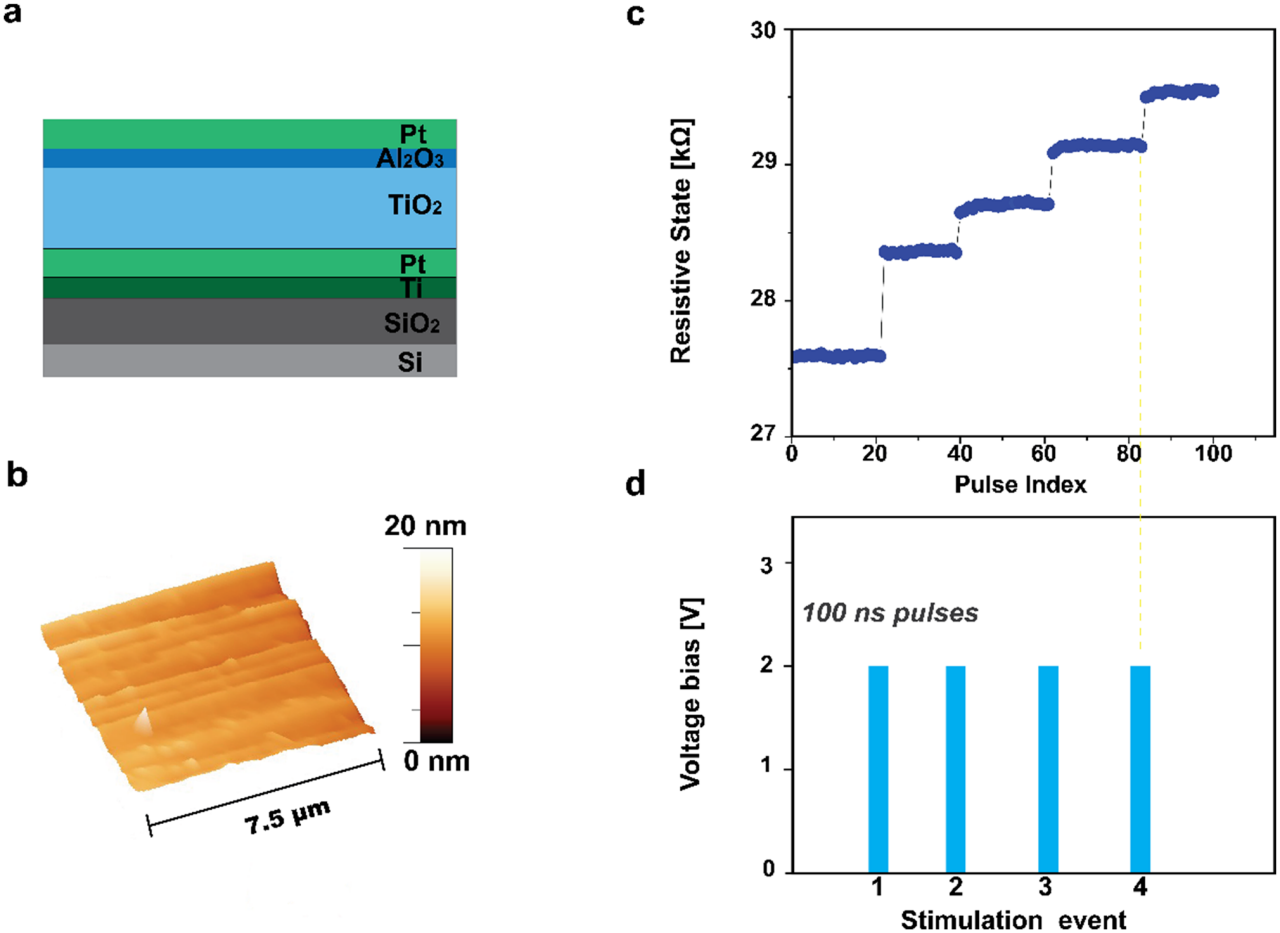
Illustration and operation of a memristor. (**a**) Schematic cross-section of the device concept comprising a Pt/TiO_2_/Al_2_O_3_/Pt metal–insulator–metal memristor. (**b**) AFM surface morphology of the device’s Pt top-electrode. (**c**) Transient response of a memristor’s state-variable RS in response to (**d**) voltage input pulses.

**Figure 2:**
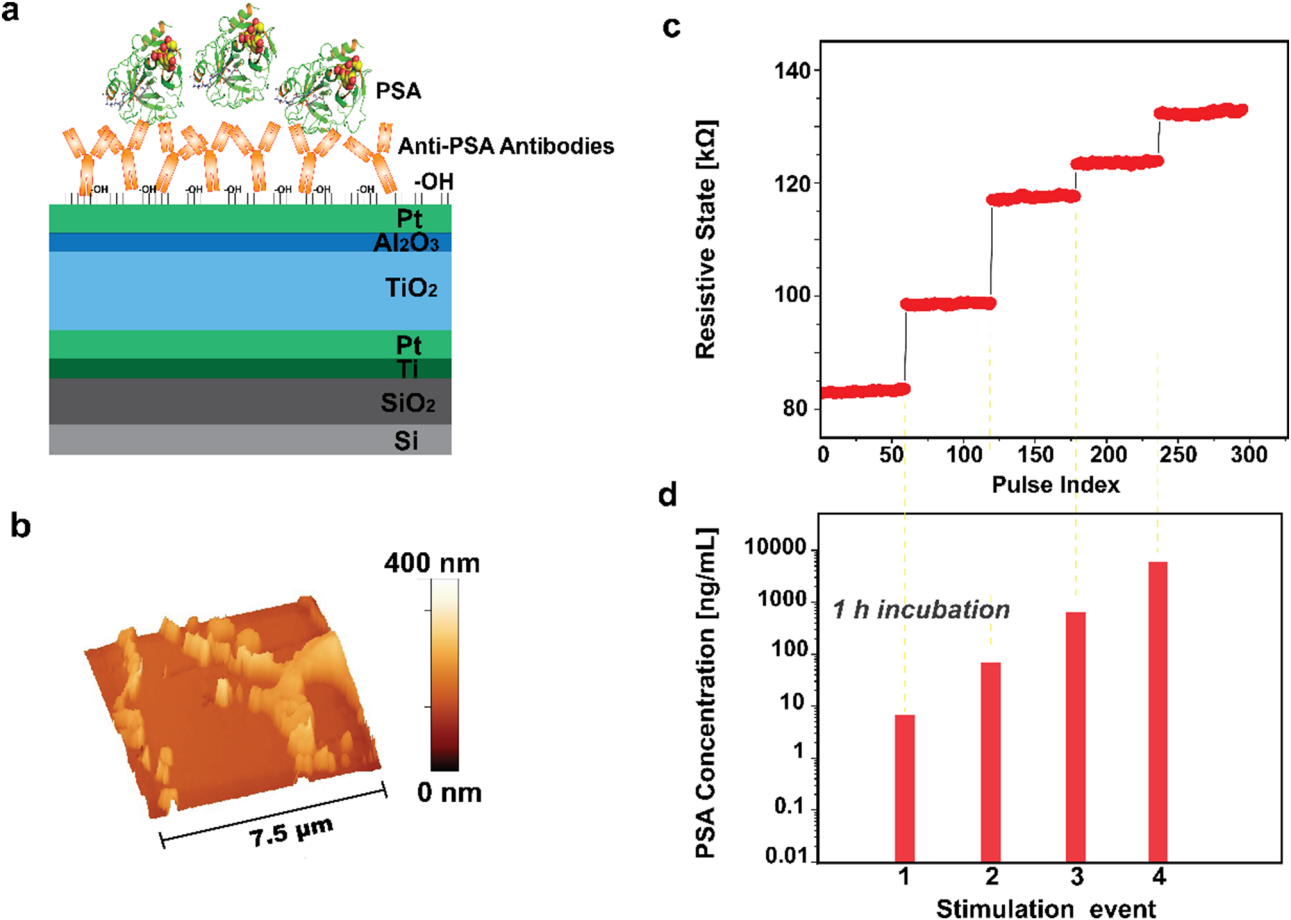
Illustration and operation of a chemical-memristor (chemristor). (**a**) Schematic cross-section of the device concept comprising a Pt/TiO_2_/Al_2_O_3_/Pt metal–insulator–metal memristor functionalized with anti-PSA antibodies. (**b**) AFM surface morphology of the device’s Pt top-electrode after the bio-functionalization. (**c**) Transient response of a chemristor’s state-variable RS in response to (**d**) bio-chemical inputs rendering distinct PSA levels.

The hereby-presented chemical-memristor (chemristor) is a modified version of a metal-oxide memristor that allows transducing chemical signals via the device’s state-variable. To achieve this, devices based on a metal–insulator–metal (MIM) architecture (Fig. 1a), are fabricated on a Silicon/Silicon dioxide (Si/SiO_2_) substrate (see Methods section), are functionalized with receptor molecules, antibodies specific to Prostate Specific Antigen (PSA) (Methods section), and then exposed to the target PSA (Fig. 2a). PSA is a 30 kDa kallikrein protein and consists one of the main biomarkers for prostate cancer (PCa)^16^, used herein as a case study for the proposed concept. Morphological analysis data obtained using Atomic Force Microscopy (AFM) before (Fig. 1b) and upon bio-functionalization (Fig. 2b) qualitatively reveal the presence of the biological substances on the surface of the memristor, that ultimately results in an increase of the surface features recorded as well as in the formation of some agglomerating patterns, due to coalesced biological molecules, that are depicted in the form of high peak wrinkles (Fig. 2b).

Pristine memristor devices react to input voltage as non-linear, thresholded, weighted integrators and as such have an inherent capability to store information as non-volatile RS transitions. Thus, the memristor instantly records the occurrence of an event as a change in RS (Fig. 1c), when subjected to an input stimulation (i.e. voltage pulse) of an amplitude that exceeds certain thresholds assessed at the desired sampling rate (Fig. 1d). In a similar fashion, the introduced chemristor device concept undergoes a memory state change (Fig. 2c) as a response to a chemical input, i.e. biomarker concentration, as showcased in Fig. 2d. Chemical and biological species such as proteins are composed of charged residues, hence demonstrating a net positive or negative charge^17^. Consequently, the additional surface charges due to proteins introduced via physical adsorption on a surface as well as to proteins bound to already immobilized receptors, and the charge neutralization/enhancement associated with the selective reaction between receptor and target molecules on the sensing surface, result in an increase/decrease in the overall net charge, and modify the effective local potential, ultimately acting equivalently to an electrical stimulus that imposes a shift in the device’s memory state. Thereafter, the target molecule is electrically detected in a label-free manner by recording the dependence of the sensor response on the applied concentration.

Therefore, while a specific state can be achieved by modulating different voltage pulse characteristics (such as number of pulses, pulse width and/or amplitude) in the presented case, it is possible to transduce distinct concentrations of PSA via analogous memory-state changes, introducing a chemical state-variable.

**Figure 3:**
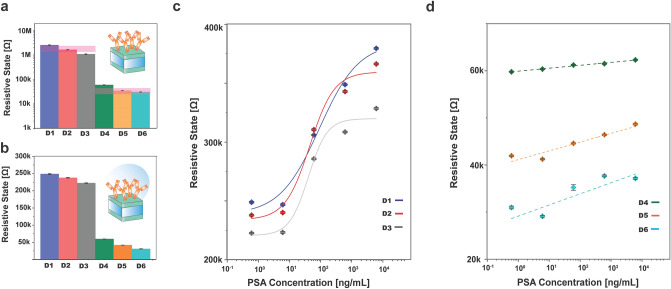
PSA sensitivity dependence on chemristors’ operating regime. (**a**) Six identical devices (D1–6) are electroformed for bringing them in to two baseline operating regimes D1–3 (I) and D4–6 (II) in dry-condition. All sensors are then exposed to a PSA solution in PBS with a mild PSA concentration of 0.6 ng/mL, resulting into a further shift in the baseline operating regimes, shown in (**b**). The PSA concentration is progressively increased (tenfold) in four stages and following incubation the RS of all sensors are recorded for all four antigen concentrations. (**c**, **d**) Depict the corresponding PSA state-dependent responses for the six transducers under consideration (D1–3 and D4–6).

In Fig. 3a, six identical devices (labeled as D1–D6), are electroformed and then functionalized with anti-PSA antibodies, demonstrating two baseline-operating regimes D1–3 (I) and D4–6 (II) respectively. At first, all devices are simultaneously exposed to a mild PSA concentration of 0.6 ng/mL in Phosphate Buffered Saline (PBS). PSA is selectively bound to the immobilized antibody since the antigenic determinant (epitope) is recognized by the paratope of the antibody ultimately forming a label-free immunoassay format. The introduction of the antigen solution results in a shift in the operating regime, as recorded in Fig. 3b. This shift can be attributed to the surface charge density modulation due to the introduction of the negatively charged PSA solution that contributes a net negative charge, masking the effect brought by the presence of antibodies^17^. The PSA concentration is then progressively increased (tenfold) in four stages and the RS of all sensors are recorded following subsequent incubation to each of the four antigen concentrations considered. The consecutive adsorption of increasing PSA concentration results in a one-way increasing trend of the devices’ RS. The obtained state-responses for the specific target are presented in Fig. 3c, d as recorded after each biomarker uptake. An average resistive level of (236.5 ± 13.2) kΩ [D1–3 (I)] and (44.2 ± 14.5) kΩ [D4–6 (II)] is first obtained for a concentration of 0.6 ng/mL in PBS and an increasing trend is acquired with the antigen uptake reaching a RS level of average value (358.2 ± 26.5) kΩ and (49.4 ± 12.6) kΩ respectively for a concentration of 6.1 μg/mL. Implementing the highest concentration of PSA, the RS level shows a total increase from the initial value of 51.4% for the operating regimes D1–3 (I) and of 13.4% for the D4–6 (II) respectively.

The introduced chemical state-variable exhibits a dependence on the initial resistance of the devices. The sensors belonging to the operating regimes D1–3 (I) demonstrate higher sensitivity when responding to externally introduced perturbations like for instance in this case the uptake of PSA. More specifically, the state-response for sensors belonging to the baseline operating regimes D4–6 (II) demonstrate a linear response with an average chemical sensitivity of (1.6 ± 0.9) kΩ/[dec.C_PSA_]. Meanwhile, the sensors belonging to the higher baseline state window [baseline operating regimes D1–3 (I)] obey a sigmoidal function while depicting a linear region (Fig. S1) with average chemical sensitivity of (68.7 ± 12.7) kΩ/[dec.C_PSA_] (Supplementary information).

We further exploit the fact that the sensitivity of the chemical memristor can be tuned in accordance to the chosen sensing operating regime, in combination with the inherent programming properties of the chemical memristors for achieving a homogeneous sensing array that comprises a uniform sensing baseline across all the individual sensing components. This can be realized by bringing all sensors of the array to a common operating regime, through normalization of the array’s sensing characteristics at the device-level, before the commencement of the sensing procedure. For achieving this hardware-based calibration, each individual chemristor is subjected to an initializing process consisting of voltage pulses of pre-specified duration and amplitude (as described in Methods), until all sensors are brought to a common operating regime.

**Figure 4:**
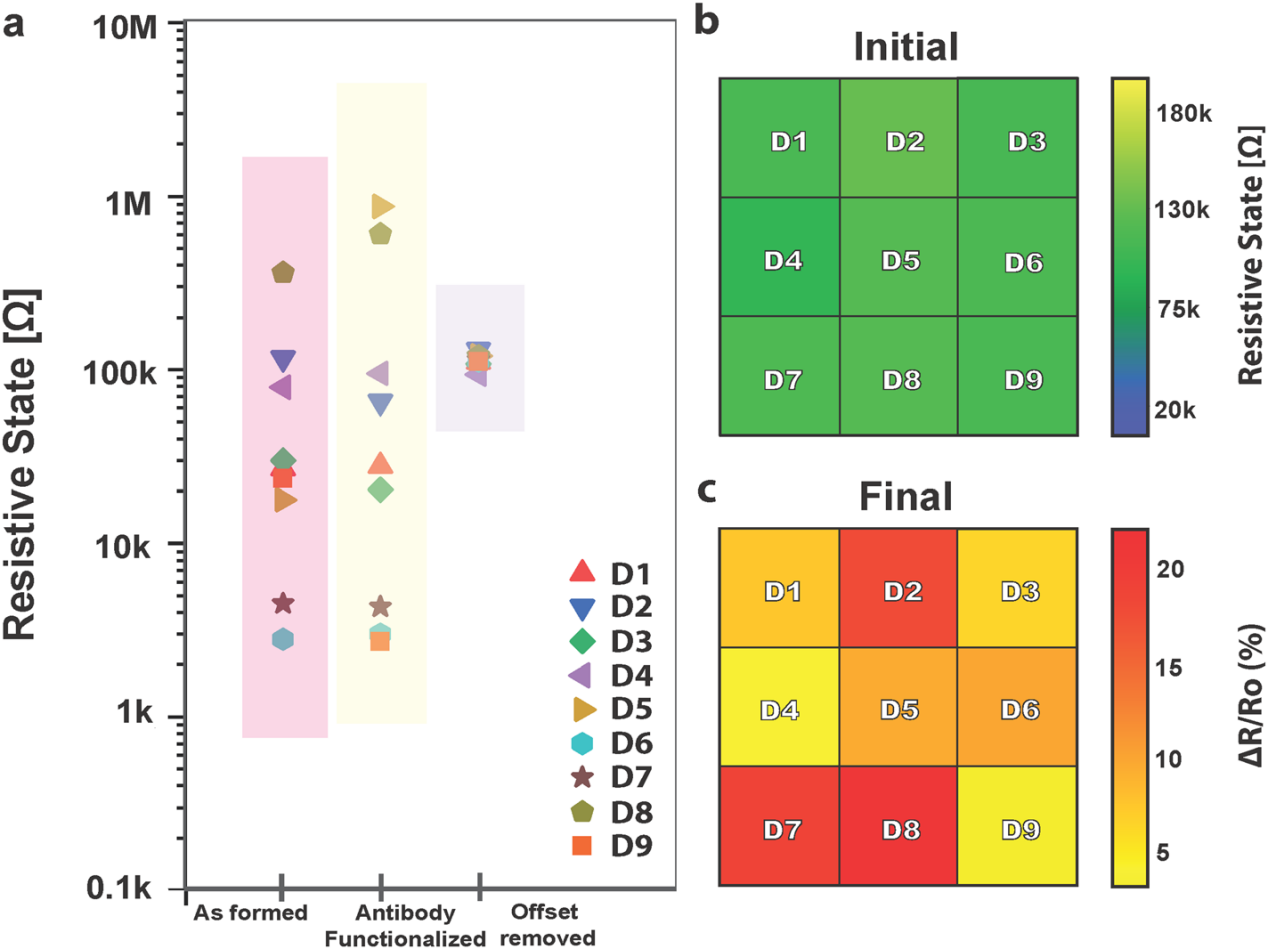
Employing device-state programmability for offset removal. (**a**) Nine identical devices (D1–D9) are electroformed for acquiring hysteretic properties. All devices are then functionalized with anti-PSA antibodies and converted to memristor-based sensing elements. Treatment with charged biological substances results into a further shift in the operating regimes. The sensors are subjected to a device-level hardware calibration, each individual chemical memristor is subjected to an initialization process comprising input programming pulses and the process is repeated until reaching the desired RS level for all the sensors under study. (**b**) RS levels achieved for the sensors (D1–D9) after the normalization procedure are represented via different pixel colors and intensities. (**c**) Corresponding PSA RS level relative change (%)-dependent responses of the nine chemical memristors (D1–D9) after exposure to different PSA concentrations as illustrated by indexed color representation.

As an example, the memory state values of a small biosensing array consisting of nine identical devices are recorded in Fig. 4a, utilizing the custom-made instrumentation^18^ (Fig. S2) and software^7^. Following bio-functionalization with anti-PSA antibodies, a further shift in the operating regimes and a larger variation among the sensing devices are observed (Fig. 4a). The array’s sensing characteristics are calibrated at the device-level, with each individual chemristor to be subjected to the initializing hardware calibration procedure, that is repeated until a common operating regime (113 ± 10) kΩ is achieved for all the sensors of the array. Figure 4b shows the calibrated memory-state values of the sensors as an output pixel-representation image.

The normalized sensor platform is then employed as an antigen sensing array, by implementing custom-made instrumentation^18^ (Fig. S2) and software^7^. Groups of individual chemristors are exposed to three PSA concentrations and their RS response is recorded, more specifically, D3, D4 and D9 to a concentration of 60.9 ng/mL, D1, D5 and D6 to 0.6 μg/mL and D2, D7 and D8 to 6.1 μg/mL. Figure 4c schematically shows the RS levels relative percentage change with respect to an initial, mild analyte concentration, operating regime, corresponding to the three employed PSA concentrations. Exposure to the lowest PSA concentration under consideration results to an average relative RS change percentage of 4.9 ± 1.6% and to a 9.2 ± 1.2% and 19.5 ± 1.8% for the medium and highest concentrations respectively.

In conclusion, the presented chemristor device concept presents a new and versatile approach for antigen-specific transduction, as the sensors’ specificity can be determined in an ad hoc manner via bio-functionalization. The inherent state-programmability attributes of the sensors can be readily used for reliably sensing a variety of biomarkers, without resourcing to external software/hardware calibration approaches; performing the calibration in-situ at device level. This capability along with the excellent scaling prospects of memristive arrays brings new prospects for realizing robust multi-panel diagnostic assays for serving the needs of modern medicine.

## Methods

### Fabrication of MIM memristors

MIM devices are realized on a 6-inch oxidized Si wafer (200 nm of dry thermal SiO_2_). Initially, 20 μm wide bottom electrodes are defined through optical lithography, electron beam evaporation of a 5 nm titanium (Ti) adhesion layer and 10 nm of platinum (Pt) and lift-off process in N-Methyl-2-pyrrolidone (NMP). Using reactive magnetron sputtering 25 nm of titanium dioxide (TiO_2−x_), as the solid electrolyte, are deposited from Ti target in a 8 sccm oxygen (O_2_) environment. Using the same process from an aluminium (Al) target, and without breaking the vacuum, a further 4 nm of aluminium oxide (Al_x_O_y_) is deposited, acting as the interface barrier layer of a final bilayer configuration. Then top 20 μm wide Pt electrodes (10 nm thickness) are defined through the same process as bottom electrodes (Fig. S3). Following the fabrication process, the wafer is diced into chips of 3 × 3 mm^2^. Each single chip, consisting of multiple MIM devices, is wire-bonded to a commercially provided ceramic quad flat J-shaped (CQFJ) chip-holder with connections suitable for the in-house memristor characterization platform that is used^18^ as it shown in Fig. S2. The MIM devices are electroformed for demonstrating hysteretic characteristics. The electroforming results to a controllable breakage of the active layer realized through the application of a pulsed voltage ramps. The biasing protocols are implemented using custom-made hardware^18^ (Fig. S2) supported by custom-made software^7^. The devices are subjected to consecutive 10 to 1000 μs pulses of negative polarity ranging from − 3 to − 12 V with a 0.25 V voltage step. Interval between pulses (interpulse time) has been kept constant at 10 ms.

### Bio-functionalization process

For the surface modification, the MIM memristive devices are treated with O_2_ plasma for 15 min (30 sccm, 99 mTorr). The plasma treatment clears any organic residues and generates free surface hydroxyl (OH)-terminating groups. Hydroxyl groups serve as surface treatment, enabling a stable chemical attachment of the biomolecules. The MIM memristors are converted to PSA-specific chemical memristors (chemristors) through a direct-adsorption surface bio-functionalization with 200 μg/mL of Anti-PSA antibody ([8A6] (ab10187) purchased by Abcam) in PBS (P4417 Sigma-Aldrich) via a 2 h incubation at room temperature. The antibody solution is introduced on the surface of the substrate via standard drop-casting using a precision micropipette (Fisher Scientific precision pipette) directly on the area of interest. After completing the incubation, the devices are gently washed using PBS solution.

### Target molecule uptake

Following the bio-functionalization process, the sensors are implemented for biomarker sensing (PSA, hereby used as a case of study). The devices are exposed to PSA (Millipore Angebot R-1939458.1; 539834 purchased from Merck) for 1 h at room temperature in PBS (P4417 Sigma-Aldrich) at the indicated concentrations belonging to the range [0.6 ng/mL–6.1 μg/mL] and achieved by following a ten-fold serial dilution protocol. The excess of antigen is removed after each biomarker update by extensive, gentle washing of the substrate surface with PBS, after each incubation.

### Electrical characterization methodology

The RS of each device is monitored by means of the custom-made instrumentation^18^ (Fig. S2) and software^7^ allowing fully automated device-by-device measurements of entire packaged arrays. Namely, following the completion of each individual step and in order to extract the RS level of each case, the devices are subjected to non-switching pulses that allow the readout without affecting the state (0.2 V read pulses set at a 1 s sampling rate) for 1 min since in this range the devices have been shown to maintain the RS for long period^7^. The measurements of the RS are performed in an intermittent way by the following sequence: MIM memristor (after the electroforming), MIM chemristor (after the bio-functionalization), and for each antigen uptake at different concentrations.

### Initializing calibration process

For the device-level hardware calibration, each individual chemical memristor is subjected to trains of input pulses using the custom-made hardware^18^ (Fig. S2) at a fixed duration in the range of 100 ns–100 μs and 1–3 V implementing steps of 50 mV. This process allows the sequential set of the memory state of the device gradually until it is stabilized at the desired level. Each cycle is alternating with RS reads at 0.2 V for recording the new RS achieved for each chemristor and the process is repeated until all the chemical memristors are brought to a common operating point.

### Surface topography characterization

Morphological AFM analysis of the structures is performed using a Bruker Atomic Force Microscope system and AFM probes, also provided by Bruker. The AFM measurements are carried out on bare samples and after the bio-modification of the surface with an Anti-PSA antibody ([8A6] (ab10187) purchased by Abcam) in PBS (P4417 Sigma-Aldrich). Tapping Mode AFM is implemented for carrying out the imaging throughout the complete characterization process, mollifying the issues of sample dragging across the surface, especially in the case where the bio-functionalized devices are considered. The data analysis is performed using WSxM 5.0 Develop 9.3 software.

## Supplementary information

Supplementary Information.

## Data Availability

The data that support the findings of this study are available from the University of Southampton institutional repository at 10.5258/SOTON/D1439.
