# Supplementary material for: Monitoring PSA levels as chemical state-variables in metal-oxide memristors
Source: Sci Rep. 2020 Sep 17;10:15281. doi: 10.1038/s41598-020-71962-3 (PMC7499304; doi:10.1038/s41598-020-71962-3)
Supplement: Supplementary file 1 — Supplementary Information. [file 41598_2020_71962_MOESM1_ESM.docx]

**Supplementary Information**

Monitoring PSA levels as chemical state-variables in metal-oxide memristors

Ioulia Tzouvadaki, Spyros Stathopoulos, Tom Abbey, Loukas Michalas, Themis Prodromakis*

Centre for Electronics Frontiers, Zepler Institute for Photonics and Nanoelectronics

University of Southampton SO17 1BJ, United Kingdom

*Corresponding author Email: [t.prodromakis@soton.ac.uk](mailto:t.prodromakis@soton.ac.uk)


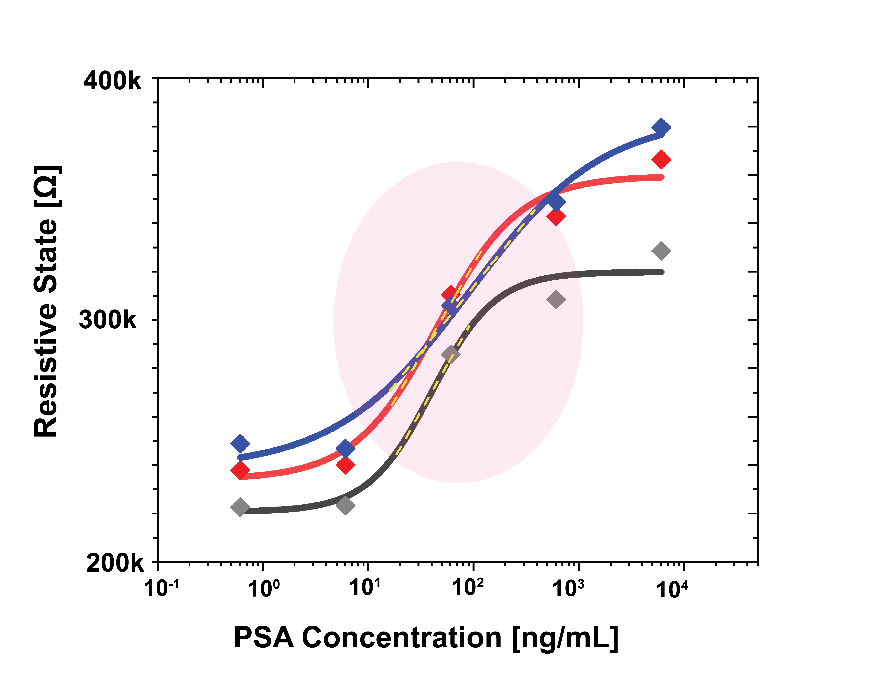


**Fig. S1**  **PSA sensitivity dependence on chemristors’ operating regime**. Corresponding PSA state-dependent responses for transducers D1-3 follow a sigmoidal function, also indicating linear regions belonging in the range of 10^1^ to 10^3^ ng/mL.

The sensors belonging to the baseline operating regimes D1-3 (I) obey a sigmoidal (Logistic dose response) function $y=A$+ $\frac{B}{1+{(x/xo)}^{p}}$ as follows:

D1 = 384.3 - $\frac{145.8}{1+{(x/xo)}^{0.68}}$ (1)

D2 = 359.6- $\frac{121.5}{1+{(x/xo)}^{1.1}}$ (2)

D3 = 319.9- $\frac{99.3}{1+{(x/xo)}^{1.4}}$ (3)

Moreover, the D1-3 (I) sensor’s responses depict a linear region that follows the equation $Y=Y_{intercept} + slope*\log X$, where the slope is defined as (kΩ /C_PSA_ decade), as hereby reported:

D1: *Y*= 205.3 + 54.29 $\log X$ (4)

D2: *Y*= 172.8 + 75.11 $\log X$ (5)

D3: *Y*= 146.8 + 76.94 $\log X$ (6)

The sensors belonging to the baseline operating regimes D4-6 (II) demonstrate linear response described as$Y=Y_{intercept} + slope*\log X$ , where the slope expresses (kΩ /C_PSA_ decade). More specifically:

D4: *Y* = 60 + 0.62 $\log X$ (7)

D5: *Y* = 41 + 1.8 $\log X$ (8)

D6: *Y* = 29 + 2.3 $\log X$ (9)


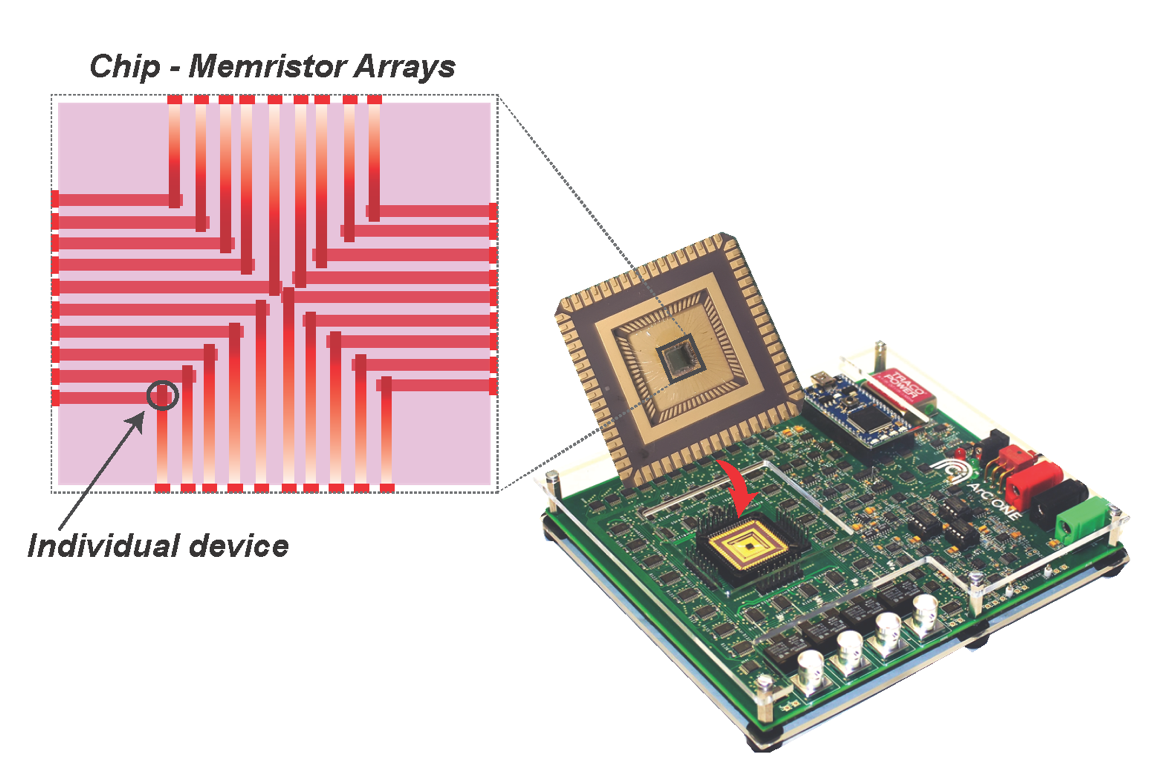


**Fig. S2 Experimental set-up.** Metal-insulator-metal (MIM) architecture forming array-based configurations. Each single chip, is wire-bonded to a commercially provided ceramic quad flat J-shaped (CQFJ) and plugged into the in-house memristor characterization platform^1^.

**Fig. S3 Device arrays structure.** Optical imaging of MIM array-based configuration comprising devices of a cross-point type of structure. The scale bar indicates 50 μm. The top and bottom electrodes as well as the resulted stand-alone device are demonstrated. Scanning Electrode Microscopy (SEM) imaging of the devices’ structure, depicting the cross-point area. is reported in^2^.

**References**

1. Berdan, R. *et al.* A μ -Controller-Based System for Interfacing Selectorless RRAM Crossbar Arrays. *IEEE Trans.Electron Devices* **62**, 2190-2196 (2015).

2. Stathopoulos, S. *et al.* Multibit memory operation of metal-oxide bi-layer memristors. *Sci. Rep.* **7**, 17532 (2017).
